# The school environment and bullying victimization among seventh graders with autism spectrum disorder: a cohort study

**DOI:** 10.1186/s13034-022-00456-z

**Published:** 2022-03-15

**Authors:** Hsin-Hui Lu, Duan-Rung Chen, An-Kuo Chou

**Affiliations:** 1 Department of Psychology, College of Medicine, Chung Shan Medical University, Taichung, Taiwan; 2 Institute of Health Behaviors and Community Sciences, National Taiwan University, Room 636, No 17, Xu-Zhou Road, Taipei 100, Taipei, Taiwan; 3 Department of Pediatrics, National Taiwan University Hsin-Chu Hospital, Hsinchu, Taiwan

**Keywords:** Bullying, Autism spectrum disorder, School environment, Adolescents

## Abstract

**Background:**

There is strong evidence to support the association between bullying and the onset of mental health conditions in students with ASD (autism spectrum disorder). In Taiwan, the seventh grade marks the first year of middle school, following elementary school. This period is also when peers tend to perform bullying behaviours to establish status among the peer group. Therefore, seventh grade is considered one of the most challenging times for students with ASD due to several changes within the school environment and the developmental changes that arise at this age. This study aims to assess the association between the school environment and bullying victimization among students with autism spectrum disorder (ASD) enrolled in regular classes in their first year of middle school.

**Methods:**

Data were obtained from the Special Needs Education Longitudinal Study database located in the Survey Research Data Archive of Academia Sinica. The analysis included one hundred eighty-four seventh graders with ASD who were in regular classes across Taiwan. The primary variables under study were whether the participants had experienced social exclusion, insults or teasing, extortion, or sexual harassment over the past semester.

**Results:**

Participants with a higher positive friendship quality (*P* = 0*.*027) and who had received more peer support upon encountering difficulties in school (*P* = 0.041) were less likely to experience social exclusion. Participants with a higher positive friendship quality (*P* = 0*.*001) and a more positive classroom learning environment (*P* = 0*.*031) were less likely to have experienced insults or teasing. However, participants with more friends were more likely to be extorted (*P* = 0*.*015) and sexually harassed (*P* = 0*.*001) than those with fewer friends. Furthermore, participants in regular classes on a part-time basis were 2.59 times more likely to report sexual harassment than those in regular classes on a full-time basis (*P* = 0*.*021).

**Conclusions:**

This study suggests that a supportive school environment reduces the likelihood that seventh-graders with ASD will be bullied. Clinicians should consider the association between the school environment and bullying victimization among adolescents with ASD in regular classes during their first year of middle school.

## Background

Adolescents with special educational needs are more likely to be victims of bullying than their classmates without such disabilities [1]. Individuals with ASD have a considerably greater risk of being victimized than their peers without ASD, a trend that applies from childhood to adulthood [2]. Studies have suggested that a combination of individual vulnerabilities in students with ASD, such as communication problems, stereotypical behaviours and interests, tendencies towards psychological distress, and aggressive behaviour, may lead to bullying victimization [3–8]. During adolescence, bullying victimization by schoolmates can result in multiple health problems, especially for individuals with autism spectrum disorder (ASD) [9, 10]. Substantial evidence supports the association between bullying victimization and the onset of mental health conditions such as panic disorder, major depression, loneliness, and social anxiety in students with ASD [5, 11, 12].

The social-ecological diathesis-stress model conceptualizes bullying as an interaction between the individual vulnerabilities (e.g., less social competence or fewer friends) of the victim and their environment [13, 14]. The association between bullying victimization and the school environment among typically developing children and adolescents comprises multiple factors, including parental engagement in school affairs, positive interpersonal interactions, peer social support, and the quality of friendships [15, 16]. The school environment has been associated with increased exposure to bullying among students with ASD [9, 17, 18]. However, whether protective measures against bullying in the school environment would be effective for Taiwanese students with ASD enrolled in regular classes during their first year of middle school has yet to be determined.

In Taiwan, the seventh grade marks the first year of middle school, following elementary school. It is considered one of the most challenging times for students with ASD due to several adjustments within the school environment and the developmental changes that arise at this age. It is also when peers tend to perform bullying behaviours to establish their peers’ status [19]. Students with ASD have significant difficulties in social interaction and communication. Their maladaptive functioning exacerbates these difficulties when they enter new social groups, particularly during the first year of the elementary-to-middle-school transition period [20]. Furthermore, the influence of environmental factors in schools on bullying victimization for seventh graders with ASD has yet to be well examined [9, 17, 18, 21]. School administrations need to implement adequate measures to protect adolescents with ASD against bullying in their first year of middle school and reduce their risk of developing future mental health problems.

The present study investigated how the school environment is associated with bullying victimization among seventh graders (aged 12–13 years) with ASD. Bullying victimization, which has been widely examined in other studies [22, 23], was analyzed in this study. We examined four types of bullying victimization separately (social exclusion, insults or teasing, extortion, and sexual harassment) and their relationships with the school environment while controlling for individual variables. The following variables of the school environment were assessed: (1) the number of friends that the students had; (2) friendship quality; (3) classroom climate; (4) receiving assistance when difficulties occurred; (5) the teacher’s attitude towards class integration; (6) parental engagement in the children’s learning; and (7) the amount of integration within the regular classes. In all analyses, the participants’ sex, body mass index (BMI), learning capabilities, and levels of psychological distress were controlled.

## Methods

### Study sample

**Fig. 1:**
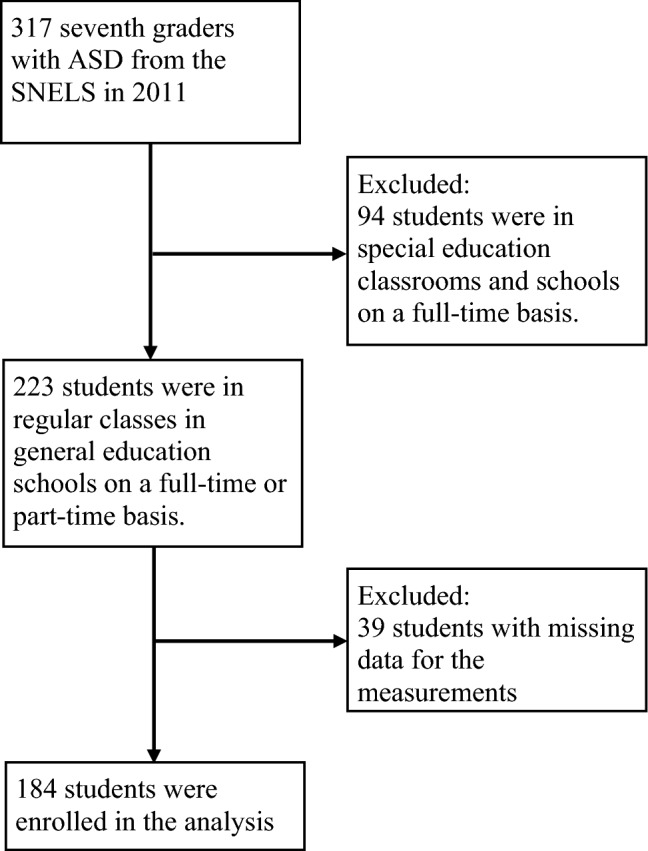
Flow chart of the selection process for study participants

The study cohort sample was taken from the Special Needs Education Longitudinal Study (SNELS) database, released in 2011 by the Survey Research Data Archive of Academia Sinica (https://srda.sinica.edu.tw/index.php). The Institutional Review Board approved the data application and analysis. In the SNELS, representative samples of students with ASD were randomly selected from a list of registrants; specifically, they were identified from medical records or records held by the Special Education Needs Committees of local governments, as described in a previous study [24]. In the SNELS, data were obtained from questionnaires administered online, which both students and teachers completed. The students completed their questionnaires independently, with the teachers providing aid if necessary. Figure 1 displays the process used for participant selection. Of the 317 seventh graders with ASD involved in the SNELS in 2011, 223 were enrolled in regular classes in general education schools. Thirty-nine seventh-graders with ASD were excluded due to a lack of data regarding the studied variables. Overall, 184 seventh graders (167 boys) with ASD were enrolled in this study.

### Types of victimization

The students were asked whether they had experienced social exclusion, insults or teasing, extortion, or sexual harassment over the past semester. Social exclusion was assessed by asking the students, “Have you felt left out by your classmates at this school?” Being insulted or teased was measured by asking the students, “Have you felt insulted or been teased by your classmates at this school?” Experiencing extortion was assessed by asking the students, “Have you experienced extortion for money at this school?” Being sexually harassed was measured by asking the students, “Has anyone touched your body inappropriately or made you feel uncomfortable at this school?” Students recorded their responses using a four-point Likert scale (1 = *never*, 2 = *seldom*, 3 = *occasionally*, and 4 = *frequently*) to indicate the frequency of bullying victimization. Answers of “never” were coded as 0, and all other responses were coded as 1.

### Student characteristics

Student characteristics comprised of the following variables: (a) sex, reported by teachers, with male and female coded as 1 and 0, respectively; (b) BMI, collected from teacher reports, was calculated as the weight in kilograms divided by the square of the height in meters; and (c) the general tendency of the students’ learning capabilities in school was collected from teacher reports. The variable of students’ learning capabilities in school was assessed using the following ten items: (1) paying attention in class; (2) complying with instructions; (3) sitting still; (4) participating in classroom discussions; (5) asking questions; (6) answering teachers’ questions; (7) focusing on schoolwork; (8) having the motivation to learn; (9) finishing homework on time, and (10) collaborating with peers to complete tasks. These items were scored on a four-point Likert scale (1 = *never*, 2 = *seldom*, 3 = *occasionally*, and 4 = *frequently*). Cronbach’s α for the ten criteria was 0.86. (d) Psychological distress was student-reported using a six-item index adapted from the Symptom Checklist-90-Revised (SCL-6) instrument, originally developed to assess a broad range of psychological symptoms [25]. The criteria were extracted based on the most frequent symptoms experienced by students receiving special education services [26]. Each of the following six dimensions comprised one item of the SCL-6: depression (feeling down or lonely); obsessive compulsion (trouble concentrating); insomnia (restlessness or disturbed sleep); anxiety (feeling tense or uptight); psychoticism (never feeling close to another person); and anger/hostility (experiencing the urge to break or smash objects or hit, injure, or harm others). The frequency of each item was rated on the same four-point scale used to assess the frequency of victimization incidents. Cronbach’s α for the SCL-6 was 0.88.

### School environment characteristics

Seven variables measured the characteristics of the school environment. The first variable was the number of friends that the students had, which was assessed using responses to “How many classmates or friends do you usually hang out with?” on a scale of 1–5 (1: *no friends*, 2: *one friend*, 3: *two or three friends*, 4: *four or five friends*, and 5: *six or more friends*). The second, friendship quality, was measured by the teachers’ responses to the question, “Is the student able to get along well with other students in the class?” on a scale of 1–5 (1: *no interaction at all*, 2: *poorly*, 3: *not very well*, 4: *acceptably well*, and 5: *very well*). The third variable, classroom climate, as measured by the teachers’ responses to the following statements, using a scale of 1–4 (1: *not at all true*, 2: *not very true*, 3: *somewhat true*, and 4: *very true*): “The majority of the students proactively participate in inter-class competitions and activities.”; “The students have positive relationships with each another”; “The students are helpful to and caring towards each another”; and “The students collaborate (i.e., discuss their homework together).”The fourth variable was whether students received assistance when difficulties occurred, which was measured by how the students responded to the question, “Are you able to get help from someone when encountering difficulties?” using a scale of 1–4 (1: *almost no one*, 2: *very few people*, 3: *some people*, and 4: *many people*). The fifth variable, the teacher’s attitude towards class integration, was assessed by the teachers’ responses to the following statements, using a scale of 1–4 (1: strongly disagree, 2: somewhat disagree, 3: somewhat agree, and 4: strongly agree): “Schools should not exclude students with disabilities from enrolment”; “Students with disabilities should be allowed to learn with their typically developing counterparts as much as possible”; “Teachers of regular classes should not neglect the needs of typically developing students in class because of the presence of students with disabilities”; “The placement of students with disabilities in the same class as typically developing students is beneficial for both groups of students”; “The teacher’s attitude is a key factor that affects the learning and adaptation of students with disabilities”; and “Teaching students with disabilities is a responsibility that is shared by teachers of both special and regular classes.” The sixth variable, parental engagement in the children’s learning, as measured by the teachers’ responses to “How would you describe the involvement of this student’s parents in their learning?” using a scale of 1–4 (1: *nonexistent*, 2: *not very high*, 3: *moderately high*, and 4: *very high*). The final variable, the amount of integration within regular classes, was measured by whether the students participated in class full-time (coded as 0) or part-time (coded as 1). Those in regular classes on a part-time basis received special education sessions at other times.

### Data analysis

All analyses were performed using the IBM SPSS Statistics program for Windows, version 22 (IBM Corp., Armonk, NY, USA). First, participant demographics and differences in the school environment for the participants who had not experienced bullying were analyzed. Continuous variables were analyzed using independent sample *t-tests*, and categorical variables were compared using the chi-square test. Next, four separate hierarchical logistic regression analyses were conducted to examine the association between aspects in the school environment and the following types of bullying victimization: social exclusion, insults or teasing, extortion, and sexual harassment. Student characteristics were input into the models to assess their initial effects on bullying victimization. After adjusting for individual factors, the variables concerning aspects of the school environment were entered and analyzed.

## Results

### Sample characteristics

Table 1 presents both the sample population’s individual and school environmental aspects. Among the 184 participants, 72.28% had experienced social exclusion, 70.11% had experienced insults or teasing, 6.52% had experienced extortion, and 28.26% had experienced sexual harassment. In contrast, only 14.13% of the participants had not experienced any bullying over the past semester. Overall, during the first year of middle school, approximately 85% of the participants had experienced at least one type of bullying, and 66.85% had experienced at least two types.

**Table 1:** Demographic characteristics of the study sample (N = 184)

	*n*	%	Mean	SD
Types of bullying victimization				
Social exclusion	133	72.28		
Insults or teasing	129	70.11		
Extortion	12	6.52		
Sexual harassment	52	28.26		
Number of types of bullying experienced				
None	26	14.13		
One type	35	19.02		
Two types	81	44.02		
Three types	39	21.20		
Four types	3	1.63		
Individual characteristics				
Sex: male	167	90.76		
Sex: female	17	9.24		
BMI			21.64	4.27
Learning capabilities			2.04	0.57
Psychological distress			2.23	0.66
School environment characteristics				
Number of friends			2.69	1.30
Friendship quality			3.66	0.87
Classroom climate			2.40	0.45
Receiving assistance when difficulties occurred			2.79	0.82
Teachers’ integration-related attitudes			2.40	0.55
Parental engagement			3.62	0.61
Integration: full-time	69	37.50		
Integration: part-time	115	62.50		

*BMI* body mass index

### Comparison of participants who had and those who had not experienced bullying victimization

As Table 2 indicates, participants who experienced social exclusion had greater psychological distress (*t* (182) = 3.90, *P* < 0.001, *d* = 0.64), fewer friends (*t* (182) = − 2.80, *P* = 0.006, *d* = 0.46), and lower positive friendship quality (*t* (134.37) = − 3.81, *P* < 0.001, *d* = 0.63) than those who had not experienced social exclusion. Moreover, their classroom environments were more negative (*t* (182) = − 2.63, *P* = 0.009, *d* = 0.43), and they received less assistance in school (*t* (182) = − 3.88, *P* < 0.001, *d* = 0.64). Participants who experienced insults or teasing reported greater psychological distress (*t* (182) = 3.34, *P* = 0.001, *d* = 0.54) and lower positive friendship quality (*t* (160.53) = − 4.24, *P* < 0.001, *d* = 0.68) than those who did not. Furthermore, their classroom environments were more negative (*t* (182) = − 3.49, *P* = 0.001, *d* = 0.56), and they received less assistance when difficulties arose in school (*t* (182) = − 2.47, *P* = 0.014, *d* = 0.40). The teachers’ attitudes were generally supportive towards class integration (*t* (182) = − 2.55, *P* = 0.011, *d* = 0.41). Compared with participants who had not experienced extortion, those who had experienced it reported having more friends (*t* (182) = 2.01, *P* = 0.046, *d* = 0.60). The participants who had experienced sexual harassment and those who had not differed in terms of the sex ratio (χ^2^ (1) = 4.63, *P* = 0.044, *d* = 0.32). Notably, participants who had experienced sexual harassment reported greater psychological distress than those who had not (*t* (182) = − 2.30, *P* = 0.023, *d* = 0.38).

**Table 2:** Chi-square and T-test results of bullying victimization by individual and school environmental characteristics (N = 184)

	Social exclusion			Insults or teasing		
	Experienced^a^ (*n* = 133)	None^a^ (*n* = 51)	*p value*^b^	Experienced^a^ (*n* = 129)	None^a^ (*n* = 55)	*p value*^b^
Individual characteristics						
Sex: male	123 (92.48)	44 (86.27)	0.254	117 (90.70)	50 (90.91)	1.000
BMI	21.76 (4.17)	21.30 (4.52)	0.505	21.72 (3.96)	21.48 (4.93)	0.730
Learning capabilities	2.01 (0.57)	2.11 (0.58)	0.293	2.02 (0.56)	2.09 (0.60)	0.449
Psychological distress	2.34 (0.62)	1.94 (0.66)	< 0.001	2.33 (0.64)	1.99 (0.64)	0.001
School environment characteristics						
Number of friends	2.53 (1.25)	3.12 (1.35)	0.006	2.64 (1.31)	2.82 (1.31)	0.386
Friendship quality	3.53 (0.92)	3.98 (0.62)	< 0.001	3.49 (0.92)	4.05 (0.56)	< 0.001
Classroom climate	2.34 (0.44)	2.53 (0.46)	0.009	2.32 (0.43)	2.57 (0.47)	0.001
Receiving assistance when difficulties occurred	2.65 (0.77)	3.16 (0.83)	< 0.001	2.70 (0.80)	3.02 (0.83)	0.014
Teachers’ integration-related attitudes	2.36 (0.55)	2.48 (0.53)	0.196	2.33 (0.54)	2.55 (0.54)	0.011
Parental engagement	3.59 (0.65)	3.71 (0.46)	0.166	3.58 (0.65)	3.71 (0.50)	0.149
Integration: full-time	46 (34.59)	23 (45.10)	0.187	44 (34.11)	25 (45.45)	0.146

	Extortion			Sexual harassment		
	Experienced^a^ (*n* = 12)	None^a^ (*n* = 172)	*p value*^b^	Experienced^a^ (*n* = 52)	None^a^ (*n* = 132)	*p value*^b^
Individual characteristics						
Sex: male	11 (91.67)	156 (90.70)	1.000	51 (98.08)	116 (87.88)	0.044
BMI	21.73 (4.92)	21.64 (4.23)	0.946	22.05 (3.89)	21.49 (4.41)	0.425
Learning capabilities	1.93 (0.68)	2.05 (0.56)	0.471	2.02 (0.55)	2.05 (0.58)	0.799
Psychological distress	2.42 (0.98)	2.22 (0.63)	0.315	2.34 (0.57)	2.19 (0.69)	0.163
School environment characteristics						
Number of friends	3.42 (1.62)	2.64 (1.27)	0.046	3.04 (1.47)	2.55 (1.21)	0.023
Friendship quality	4.00 (0.60)	3.63 (0.88)	0.069	3.58 (0.89)	3.69 (0.86)	0.429
Classroom climate	2.52 (0.51)	2.39 (0.45)	0.321	2.40 (0.47)	2.40 (0.45)	0.945
Receiving assistance when difficulties occurred	3.00 (0.85)	2.78 (0.82)	0.367	2.79 (0.85)	2.80 (0.81)	0.958
Teachers’ integration-related attitudes	2.50 (0.52)	2.39 (0.55)	0.497	2.35 (0.62)	2.41 (0.52)	0.503
Parental engagement	3.58 (0.52)	3.62 (0.61)	0.831	3.58 (0.72)	3.64 (0.56)	0.551
Integration: full time	3 (25.00)	66 (38.37)	0.539	14 (26.92)	55 (41.67)	0.090

*BMI* body mass index

^a^Data are presented as the *n* (%) or mean (standard deviation)

^b^Chi-square tests or *t tests*

### The school environment characteristics of bullying victimization

Table 3 presents the determinants of experiencing social exclusion, insults or teasing, extortion, and sexual harassment over the past semester (Models 1–4).

**Table 3:** Final hierarchical logistic regression of bullying victimization (N = 184)

AORs [95% CIs]	Social exclusion		Insults or teasing		Extortion		Sexual harassment	
Individual characteristics								
Sex: male ^Ref = female^	1.47	[0.44, 4.90]	0.67	[0.19, 2.44]	1.02	[0.11, 9.40]	8.44*	[1.02, 69.91]
BMI	1.05	[0.96, 1.15]	1.01	[0.93, 1.11]	0.99	[0.84, 1.16]	1.02	[0.94, 1.11]
Learning capabilities	1.32	[0.62, 2.81]	1.54	[0.73, 3.26]	0.57	[0.17, 1.95]	1.15	[0.57, 2.29]
Psychological distress	2.66**	[1.37, 5.17]	2.59**	[1.35, 4.95]	2.24	[0.81, 6.15]	1.72	[0.98, 3.04]
School environment characteristics								
Number of friends	0.83	[0.61, 1.14]	1.06	[0.78, 1.44]	1.95*	[1.14, 3.35]	1.66**	[1.22, 2.25]
Friendship quality	0.55*	[0.32, 0.93]	0.40**	[0.24, 0.70]	1.55	[0.56, 4.27]	0.73	[0.46, 1.14]
Classroom climate	0.52	[0.22, 1.24]	0.39*	[0.17, 0.92]	2.58	[0.52, 12.65]	1.46	[0.65, 3.30]
Receiving assistance when difficulties occurred	0.57*	[0.33, 0.98]	0.87	[0.52, 1.44]	1.09	[0.47, 2.53]	0.90	[0.56, 1.44]
Teachers’ integration-related attitudes	1.07	[0.53, 2.17]	0.69	[0.34, 1.41]	1.46	[0.40, 5.32]	1.03	[0.52, 2.03]
Parental engagement	0.81	[0.41, 1.59]	0.84	[0.43, 1.63]	0.78	[0.28, 2.18]	0.92	[0.52, 1.64]
Integration: part time ^Ref = full time^	1.25	[0.55, 2.84]	1.71	[0.77, 3.83]	1.95	[0.40, 9.52]	2.59*	[1.16, 5.80]
− 2 Log likelihood	177.63		182.76		87.33		194.95	
Omnibus tests	χ^2^_(7)_ = 23.00, *P* = .002		χ^2^_(7)_ = 30.16, *P* < .001		χ^2^_(7)_ = 1.39, *P* = .846		χ^2^_(7)_ = 16.15, *P* = .024	

**P < .01

*P < .05

*BMI* body mass index

#### Social exclusion

Participants who had higher positive friendship quality (adjusted odds ratio [AOR] = 0.55, 95% confidence interval [CI] 0.32–0.93, *P* = 0.027) or had received more assistance when encountering difficulties in school (AOR = 0.57, 95% CI 0.33–0.98, *P* = 0.041) were less likely to experience social exclusion. Additionally, participants who reported having greater psychological distress experienced more social exclusion than those who did not (AOR = 2.66, 95% CI 1.37–5.17, *P* = 0.004).

#### Insults or teasing

Participants who had higher positive friendship quality (AOR = 0.40, 95% CI 0.23–0.70, *P* = 0.001) or were in more positive classroom learning environments (AOR = 0.39, 95% CI 0.17–0.92, *P* = 0.031) were less likely to experience insults or teasing. Furthermore, participants who reported greater psychological distress experienced insults or teasing more often than those who had less or no distress (AOR = 2.59, 95% CI 1.35–4.95, *P* = 0.004).

#### Extortion

Participants who reported having more friends (AOR = 1.95, 95% CI 1.14–3.35, *P* = 0.015) were more likely to have experienced extortion than those with fewer friends.

#### Sexual harassment

Participants who reported having more friends (AOR = 1.66, 95% CI 1.22–2.25, *P* = 0.001) were more likely to have been sexually harassed than those with fewer friends. Furthermore, students in regular classes on a part-time basis were 2.59 times more likely to report sexual harassment than those in regular classes on a full-time basis (AOR = 2.59, 95% CI 1.16–5.80, *P* = 0.021). Additionally, male students were 8.44 times more likely to report sexual harassment than female students (AOR = 8.44, 95% CI 1.02–69.92, *P* = 0.048).

## Discussion

This study examined how students’ characteristics and the aspects of the school environment are associated with experiences of being bullied among adolescents with ASD in the first year of middle school. To the best of our knowledge, this is the first study to examine the possible association between bullying victimization and classroom environments using a sample of seventh graders with ASD. The results revealed that seventh-graders with ASD who had higher-quality friendships or received assistance when encountering difficulties in school had a lower risk of being socially excluded. Regarding insults or teasing, the school environment was shown to play an essential role alongside the positive impact of the adolescents’ friendships. Students with ASD who experienced high social cohesion, harmony, and assistance in regular classes had a lower risk of being insulted or teased by their classmates. This finding indicates that social acceptance by fellow students could reduce the risk of students with ASD experiencing victimization and suggests that a positive classroom environment is an essential factor that protects against social exclusion and insults or teasing.

Hebron and Humphrey (2014) found that positive peer relationships are associated with lower bullying victimization levels. They argued that positive peer relationships could provide a friendly environment for learning social skills and are essential for protection against victimization [17]. The current findings are consistent with those from studies with typically developing adolescents, which suggest that peer victimization is less likely to occur in a classroom environment comprising positive, warm, and supportive peer relationships [27, 28]. Thus, efforts to reduce bullying should emphasize empathy among students in regular class settings.

Positive friendship quality, receiving social aid while at school, and a positive classroom learning environment decrease the risk of being bullied. Typically developing students with higher-quality friendships are likely to accept social support and connectedness and embrace diversity at school: social capital’s core elements [29]. Building social capital, a sense of community, and positive interpersonal and intergroup relationships at school are essential for preventing bullying [30]. Additionally, a friendly and supportive school environment that guides students with an unconditionally positive atmosphere also enhances social capital by promoting the belief that people are trustworthy, fair in their actions, and helpful when needed [31]. Whether anti-bullying efforts only focus on a single type of bullying, building social capital at schools is essential to developing social-ecological interventions to prevent the bullying of adolescents with ASD.

However, participants with more friends were more likely to experience extortion or sexual harassment than those with fewer friends. This result contrasts with previous studies that found that students with ASD who had fewer friends at school were more likely to be victimized [3, 18]. This suggests that having friends does not necessarily guarantee protection and support, as some friends can be aggressive or abusive. Studies have indicated that a substantial proportion of bullying events occur within the boundaries of perceived friendships [32, 33]. Additionally, regarding sexual harassment, a discrepancy often exists between the offenders’ and victims’ interpretations of behavioural intentions. A victim’s perception may be more critical than an aggressor’s intent in identifying whether victimization has occurred. Finally, when individual characteristics were controlled for (i.e., sex, BMI, learning capabilities, and psychological distress), participants in regular classes on a part-time basis were more likely to be victims of sexual harassment than those in regular classes on a full-time basis. However, in general, the association between the amount of integration in regular classes and the sexual harassment of students with ASD is unclear and warrants further investigation.

Regarding the individual characteristics related to vulnerability, participants with more significant levels of psychological distress were more likely to experience social exclusion and insults or teasing than those with lower levels of psychological distress. Previous studies have shown that depression, anxiety, and stress are significantly associated with bullying victimization among adolescents. The elevated levels of psychological distress resulting from these conditions constitute risk factors for bullying victimization [34–36]. In this study, male participants were more likely to have experienced sexual harassment than female participants. However, of the current sample of 184 students, only 17 were female. Therefore, there may not have been sufficient data to properly analyze the actual differences in experiences between boys and girls. Future studies should compare the risk of sexual harassment victimization among male and female adolescents with ASD and delineate the mechanisms that underlie any sex differences.

In conclusion, the longitudinal/accumulated effects of bullying on adolescents with ASD seen during the first year of middle school represent pressing issues. However, the present study only examined the association between bullying victimization and social exclusion, insults or teasing, extortion, and sexual harassment in the school environment. Future studies are still needed regarding the potential association between other types of bullying and the school environment and bullying studies outside of schools/in special education classes [13].

### Limitations

Instead of self-reported bullying victimization, there is a significant need to use reports from multiple informants, including parents and teachers [37, 38]. In Taiwan, middle-school homeroom teachers do not typically remain in the classroom throughout the day, thus preventing these teachers from observing student interactions. Furthermore, adolescent students tend to be reluctant to tell their parents about their experiences in school. As previously mentioned, a victim’s perception, rather than an aggressor’s intent, maybe more crucial in identifying victimization. Therefore, this study’s self-reported experiences of bullying victimization are considered valid. However, bias or faulty awareness was likely present in these interpersonal interactions self-reports. Future research with this population should further identify bias in student reports and collect data from multiple informants.

## Conclusions

Within this study cohort, approximately 85% of the seventh graders with ASD had experienced at least one type of bullying. The data suggest that students with ASD are likely to become victims of bullying while adapting to new schools and during the period when peers form connections and adopt bullying behaviours to establish status. These results highlight the need for intervention and proactive prevention strategies against bullying to help students with ASD participate in regular classes during the first year of middle school. Moreover, the school environment strongly contributed to the risks of being victimized, depending on the kind of bullying. Unexpectedly, adolescents with ASD enrolled in a regular class setting had an elevated risk of bullying victimization. This finding underlines the complex mechanisms of bullying victimization in school environments.

Social support, positive friendships, and a positive learning environment were indicated among students as protective factors against bullying victimization. This finding can serve as reference data for developing future anti-bullying programs designed for school settings. More programs that focus on inclusion and diversity are needed to aid adolescents with ASD who participate in regular class settings and prevent their likelihood of suffering from bullying. Positive school environments have been shown to help reduce the frequency of bullying behaviours [39, 40]. Implementing more steps towards this goal is expected to facilitate the successful integration of students with ASD into regular class settings.

## Data Availability

The data that support the findings of this study are available in the Special Needs Education Longitudinal Study (SNELS) database, released from the Survey Research Data Archive of Academia Sinica. Restrictions apply to the availability of these data used under license for the current study and are not publicly available. Data are, however, available from the authors upon reasonable request and with permission of the Survey Research Data Archive of Academia Sinica.

## Abbreviations

ASD: Autism spectrum disorder
SCL-6: Symptom checklist-90-revised
SNELS: Special needs education longitudinal study
